# Low-Cost Microwave-Assisted Partial Pseudomorphic Transformation of Biogenic Silica

**DOI:** 10.3389/fchem.2019.00575

**Published:** 2019-08-13

**Authors:** Denise Schneider, Ralf Kircheis, Susan Wassersleben, Wolf-Dietrich Einicke, Roger Gläser, Dirk Enke

**Affiliations:** Institute of Chemical Technology, Universität Leipzig, Leipzig, Germany

**Keywords:** biogenic silica, high porosity, partial pseudomorphic transformation, microwave synthesis, green chemistry

## Abstract

This work introduces a cost and time efficient procedure to specifically increase mesopore volume and specific surface area of biogenic silica (specific surface area: 147 m^2^ g^−1^ and mesopore volume: 0.23 cm^3^ g^−1^) to make it suitable for applications in adsorption or as catalyst support. The target values were a specific surface area of ~500 m^2^ g^−1^ and a mesopore volume of ~0.40–0.50 cm^3^ g^−1^ as these values are industrially relevant and are reached by potential concurring products such as precipitated silica, silica gel, and fumed silica. The applied process of partial pseudomorphic transformation was carried out as a single reaction step in a microwave reactor instead of commonly used convective heating. In addition, the conventionally used surfactant cetyltrimethylammonium bromide (CTABr) was substituted by the low-cost surfactant (Arquad® 16-29, cetyltrimethylammonium chloride (CTACl) aqueous solution). The influence of microwave heating, type of surfactant as well as the concentration of NaOH and CTACl on the textural and structural properties of the modified biogenic silica was investigated using nitrogen adsorption as well as scanning and transmission electron microscopy. The results show that the textural parameters of the modified biogenic silica can be exactly controlled by the amount of NaOH in the reaction solution. By variation of the NaOH concentration, specific surface areas in the range of 215–1,001 m^2^ g^−1^ and mesopore volumes of 0.25–0.56 cm^3^ g^−1^ were achieved after reaction at 393 K for 10 min. The presented microwave route using the low-cost surfactant solution decreases the reaction time by 99% and as shown in an example for German prices, lowers the costs for the surfactant by 76–99%.

## Introduction

As environmental awareness is moving into the focus, sustainable, regionally available, and CO_2_ neutral sources for the synthesis of porous materials are highly demanded. Porous biogenic silica can be obtained by a valorization process from different types of Si-containing biomass and agricultural waste products like rice, spelt and oat husk, horsetail, cereal remnant pellets, wheat, and rice straw (Alyosef et al., 2013, 2015; Schneider et al., 2018). It is an attractive material with respect to ecological, economic, and environmental reasons. The production of biogenic silica from agricultural waste products combines many benefits. Agricultural waste products are a cheap and abundant resource as an alternative for products like precipitated silica, silica gel, and fumed silica. The usage of agricultural wastes solves disposal problems, there are no competing interests with the food sector and the combustion step during the production of biogenic silica can be coupled with energy/steam production.

The porous character of biogenic silica enables an application, for instance as low-cost, non-conventional adsorbent and viable alternative to expensive activated carbon, e.g., for the treatment of effluents to remove toxic metal ions or dyes. As described in literature, biogenic silica (e.g., rice husk ash) from industrial scale/without pre-treatment before combustion exhibits specific surface areas of 20–130 m^2^ g^−1^ and mesopore volumes of 0.04–0.21 cm^3^ g^−1^ (Lataye et al., 2008; Foo and Hameed, 2009; Lakshmi et al., 2009). However, for an application in catalysis or adsorption, it is favorable to use materials with higher specific surface areas (~500 m^2^ g^−1^) and mesopore volumes (~0.40–0.50 cm^3^ g^−1^). Previous studies already showed the synthesis of adsorbents with high specific surface areas and mesopore volumes based on agricultural or industrial waste products acting as a cheap precursor, as conventional Si sources such as n-alkoxysilanes, alkali silicates, and fumed silica are produced in highly energy consuming and therefore costly processes. Hence, a conversion of both agricultural and industrial waste products into materials with improved textural features e.g., activated carbons (Daud and Ali, 2004; Patel, 2004; Mohan et al., 2008; Budinova et al., 2009; Yeletsky et al., 2009; Dutta et al., 2011; Arami-Niya et al., 2012; Hesas et al., 2013; Dasgupta et al., 2015; Tsyntsarski et al., 2015; Yahya et al., 2015; Gonsalvesh et al., 2016), ZSM-5 (Chareonpanich et al., 2004; Vempati et al., 2006; Kordatos et al., 2008; Bhagiyalakshmi et al., 2010; Pimprom et al., 2015; Ravandi et al., 2018; Zhang et al., 2018), SBA-15/-16 (Kumar et al., 2001; Chandrasekar et al., 2008, 2009; Renuka et al., 2013), or MCM-22/-41/-48 (Endud and Wong, 2007; Bhagiyalakshmi et al., 2010; Cheng et al., 2012; Alyosef et al., 2014; Li et al., 2016) was suggested in literature.

This contribution aims to conduct a partial transformation of meso/macroporous biogenic silica into Micelle-templated silicas (MTS) (namely MCM-41 like materials). There are different approaches in literature to synthesize MCM-41 based on agricultural and industrial waste products. Table 1 summarizes an overview of the starting materials, the synthesis conditions, and the resulting textural parameters (specific surface area and mesopore volume) of selected literature. As it can be deducted from the table, the syntheses have a duration of at least 3 h, with the majority taking 1 to 5 days. The resulting adsorbents are characterized by specific surface areas of 600–1,300 m^2^ g^−1^ and mesopore volumes in the range 0.5–1.1 cm3 g^−1^.

**Table 1:** Synthesis conditions and textural properties of MCM-41 materials derived from agricultural and industrial waste products.

**Starting material**	**Synthesis conditions**	***S_***BET***_*/m^**2**^ g^**−1**^**	***V_***P***_*/cm^**3**^ g^**−1**^**	**Refs**
Sedge ash	373 K/3 d	~1,200	0.98	Ghorbani et al., 2013
Rice husk ash	298 K/2 d	~600	0.49	Renuka et al., 2013
Rice husk ash	298 K/2 d	~700	0.87	Chiarakorn et al., 2007
Power plant bottom ash	373 K/2 d	~800	~0.70	Chandrasekar et al., 2008
Coal fly ash	373 K/5 d	~700	n. d.	Misran et al., 2007
Coal fly ash	353 K/3.5 h	~1,200	~0.95	Yan et al., 2016
Coal fly ash	298 K/1 d	400–1,100	0.60–0.90	Hui and Chao, 2006
Miscanthus bottom ash	298 K/1 d	~1,000	1.09	Dodson et al., 2013
Acid leaching residue of coal gasification slag	298 K/13 h	~1,300	0.83	Li et al., 2016
Rice husk ash	373 K/4 d	~1,100	0.96	Bhagiyalakshmi et al., 2010

*Refs, references; n. d., not determined*.

However, in these studies, the silica contained in the waste products is dissolved to result in sodium silicate solutions which were then used for templated mesoporous silica synthesis. A direct synthesis with a single reaction step based on the principle of pseudomorphic transformation for the synthesis of, e.g., MCM-41/-48 materials is more viable for economic and ecologic reasons. For example, the synthesis of MCM-41/-48 via pseudomorphic transformation of rice husk ash has already been shown in our previous work (Alyosef et al., 2014). The complete transformation of the starting materials into MCM-41/-48 took 6 days and was carried out under hydrothermal conditions (autoclave, 393 K) using convective heating to result in materials with specific surface areas of about 1,200 m^2^ g^−1^ and mesopore volumes of ~0.8–1.0 cm^3^ g^−1^ (Alyosef et al., 2014).

The concept of pseudomorphic transformation was first introduced by the group of Galarneau (Martin et al., 2002). During the transformation, a dissolution-reprecipitation mechanism of the silica precursor occurs. This is usually carried out using a solution of an alkaline medium, e.g., NaOH, and a quaternary ammonium surfactant (C_n_H_2n+1_-N${\left({\text{CH}}_{3}\right)}_{3}^{+}$ X^−^, *n* = 8…22, X^−^ = Cl^−^, Br^−^, OH^−^) which may form surfactant micelles. The chain length determines the size of the micelles and therefore the pore width of the resulting material after transformation (Beck et al., 1992). Usually, cetyltrimethylammonium compounds, e.g., CTABr, are used as structure directing agent (SDA). Under alkaline conditions, the silica of the starting material is dissolved and the SDA acts simultaneously as a template for the self-assembly of the silica species. There are many different mechanisms discussed in previous studies (Beck et al., 1992; Kresge et al., 1992; Chen et al., 1993; Monnier et al., 1993; Steel et al., 1994; Firouzi et al., 1995). With increasing surfactant concentration, the form of the assemblies changes from spherical micelles to rod-like micelles (Cai et al., 1999), hexagonal structures and molecular arrays or supramolecular arrays for MCM-41/-48 at higher concentrations. For achieving a full transformation, molar ratios of NaOH/CTABr 2.5: 1 for LiChrospher® 100 and Nucleosil® 100-5 (Galarneau et al., 2006) or 1: 1 for controlled porous glass (CPG) (Einicke et al., 2013b; Uhlig et al., 2013) and biogenic silica (Alyosef et al., 2014) were applied in literature. Since a ratio of 1:1 has proven to be suitable for biogenic silica, this ratio was used as a starting point in the present study. However, there are two disadvantages of this route: the high costs of the commonly used surfactant CTABr and the long reaction time using convective heating in an oven.

To overcome these drawbacks, this work addresses a partial pseudomorphic transformation in a rapid microwave synthesis. Microwave synthesis of (Al-)MCM-41 was already shown in literature by Wu and Bein (1996) and Park et al. (1998) using precipitated and colloidal silica as a precursor. For the first time in literature, this work applies a low-cost route using an inexpensive commercial detergent solution containing CTACl (Arquad® 16-29) as a substitute SDA and NaOH for the dissolving function. The use of CTACl rather than CTABr has already been reported in literature and suggest that a replacement of the anion of the tenside is feasible (Qiao et al., 2009; Sandoval-Díaz et al., 2017).

The aim of this work is to increase the specific surface area of biogenic silica by a factor of three to ~500 m^2^ g^−1^ and double the mesopore volume to ~0.40–0.50 cm^3^ g^−1^ as these values are industrially relevant and are reached by potential concurring products such as precipitated silica, silica gel and fumed silica (Ferch, 1976). In this study, the increase of specific surface area and mesopore volume should be carried out in a low-cost, rapid synthesis without performing a complete pseudomorphic transformation. Galarneau et al. (2009) already conducted a partial pseudomorphic transformation to perform a surface roughening of silica gels for HPLC applications. The synthesis took 3 to 20 h and was limited to the surface of the macropores (*d*_*P*_ = 80 nm). The resulting materials had specific surface areas of 94–457 m^2^ g^−1^ and a surface roughness of 10 nm width and depth.

During the experimental part of the investigations, biogenic silica based on rarely studied spelt husks was prepared. Firstly, citric acid was used to perform the leaching of the biomass to remove inorganic impurities by chelating metal ions (Umeda and Kondoh, 2010; Alyosef et al., 2013). Otherwise, the formation of crystalline silica will be enhanced by alkali ions like K^+^, Na^+^, and carbon might be entrapped in the material during burning, which would complicate the pseudomorphic transformation (Venezia et al., 2001; Umeda and Kondoh, 2010). Citric acid can be produced sustainably using the fungus *aspergillus niger* and does not release hazardous ions or gases which makes it ecologically superior to inorganic acids (Vandenberghe et al., 2000). Secondly, the organic components of the leached husks were removed by burning to obtain the biogenic silica ash. The partial pseudomorphic transformation was conducted in a microwave reactor using NaOH and CTACl in varying concentrations to study their influence on the resulting textural properties. All samples were characterized by nitrogen sorption. Furthermore, selected samples were investigated by scanning and transmission electron microscopy (SEM/TEM).

The novelties of this work can be summarized as follows:

- The conduction of a partial pseudomorphic transformation rather than a full synthesis of MCM-41 material to increase the textural parameters in a single reaction step using biogenic silica derived from spelt husk.
- The use of a low-cost detergent solution containing CTACl (as shown in an example for Germany, the price can be decreased by 76–99% as compared to the route using CTABr).
- The shortage of the reaction time by using microwave synthesis (99% of reaction time saved as compared to the route using convectional heating).

## Experimental

### Materials

Spelt husks were provided by Bayerischer Müllerbund e.V., Germany. HPLC silica gel was taken from a PREPPAK®500 Silica column, Millipore corporation, Waters Chromatography Division, Milford, USA, and Aerosil® 90 was purchased from Evonik, Hanau, Germany.

The used chemicals were citric acid (C_6_H_8_${\text{O}}_{7}^{*}$H_2_O, technical grade, Citrique Belge, Tienen, Belgium) for leaching of the spelt husks; sodium hydroxide (NaOH, Merck KGaA, Darmstadt, Germany), cetyltrimethylammonium bromide (Fluka Chemie GmbH, Buchs, Switzerland), and cetyltrimethylammonium chloride (Arquad^®^ 16-29, Akzo Nobel, Stenungsund, Sweden) (29 wt.% solution of CTACl in water) for partial pseudomorphic transformation. In all cases, deionized water was used.

### Methods

Biogenic silica was produced in two steps. Hundred grams of spelt husks were subjected to 1,300 ml of a 7 wt.% citric acid solution (solid-to-liquid ratio 1:13) and agitated in a four-necked round-bottom flask at 450 rpm (mechanical stirrer: Heidolph RZR 2021, Schwabach, Germany) and 353 K for 2 h. After filtration, the specimens were washed with water and dried overnight at 323 K in a drying oven (WTB Binder, Tuttlingen, Germany). In the second step, the treated husk samples were burned as reported in Schneider et al. (2018).

As a reference, partial pseudomorphic transformation was performed in a drying oven using convective heating. Twenty-one milliliter of an aqueous solution of 0.09 M NaOH and 0.09 M CTACl were added to 1 g biogenic silica and hydrothermally treated for 24 h at 393 K in a PTFE bottle (oven: VWR VENTI-Line, Leuven, Belgium). Partial pseudomorphic transformation in the microwave was carried out in a PTFE microwave vessel containing 1 g of biogenic silica and 21 ml of an aqueous solution containing 0.05–0.30 M NaOH and 0.01–0.09 M CTACl. In addition, a reference of the conventional route was carried out using a solution 0.09 M NaOH and 0.09 M CTABr for microwave reaction. The microwave synthesis (speedwave 4, Berghof Products + Instruments GmbH, Eningen, Germany) was performed at 393 K (directly determined by a DIRC thermometer for each vessel) for 10 min at 360 W. After partial transformation, the samples were washed thrice with 50 ml water and dried at 323 K for 24 h (oven: WTB Binder, Tuttlingen, Germany). The surfactant was removed by calcination using the following temperature program with a heating rate of 600 K h^−1^: 473 K/2 h, 673 K/2 h, 813 K/2 h.

### Characterization

Elemental analysis was performed by XRF (S4 Explorer, WDXRF, Bruker, Karlsruhe, Germany) after preparation according to Schneider et al. (2018). The amorphous content of the ash was determined by X-Ray powder diffraction (XRD, D8 DISCOVER, Bruker, Germany equipped with a VÅNTEC-500 2D detector) using Cu-Kα 1 and 2 radiation at 40 kV and 40 mA. 5 frames were recorded with a measurement time of 10 min per frame. Textural properties were determined by nitrogen sorption (ASAP 2000, Micromeritics, Norcross, GA, USA) at 77 K after activating the samples for 12 h under ultrahigh vacuum at 523 K. The total mesopore volume was calculated at *p/p*_0_ = 0.995. The specific surface area was determined using the model of Brunauer, Emmett, and Teller (BET) in a relative pressure range of *p/p*_0_ = 0.05–0.30 (Brunauer et al., 1938). The pore width distribution was calculated by the non-local density functional theory (NLDFT) using the software Quantachrome ASiQwin. Scanning electron microscopic (SEM) pictures were recorded at 10 kV (FEI Nano CAB 2000, Thermo Fisher Scientific Inc., Massachusetts, USA) after coating the samples with a gold layer. Transmission electron microscopy (TEM) images were obtained using an accelerating voltage of 100 kV (JEOL JEM 1010, Joel Korea ltd., South Korea). Small Angle X-Ray scattering (SAXS, X‘pert Pro, Philips/PANalytical, Almelo, Netherlands) was carried out using Cu-Kα radiation (λ = 1.54 nm) with *2*θ = 0.5-5.5°.

## Results and Discussion

### Characterization of Starting Material and Influence of Microwave Heating/Surfactant on Textural Properties

**Figure 1:**
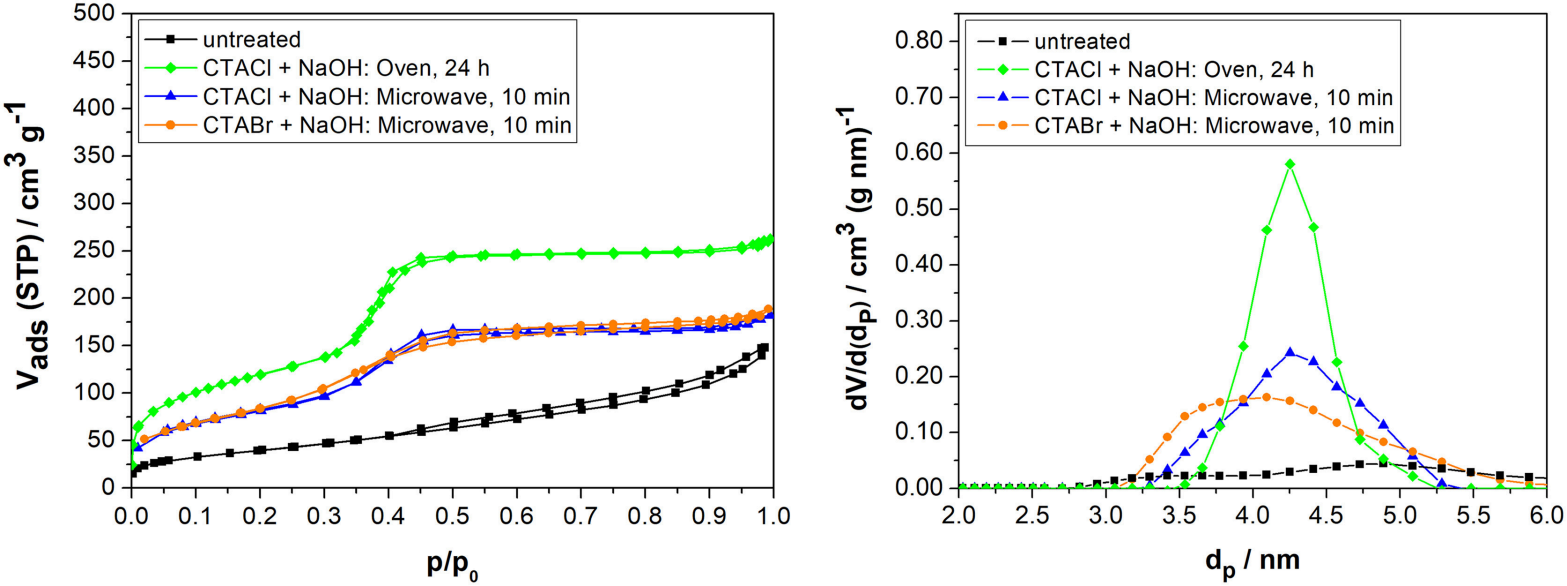
Nitrogen sorption isotherms (left) and pore width distributions (NLDFT method, right) of biogenic silica before and after partial transformation in the oven for 24 h (convective heating), and in the microwave for 10 min at 393 K with either 0.09 M CTACl and 0.09 M NaOH, or 0.09 M CTABr and 0.09 M NaOH.

**Figure 2:**
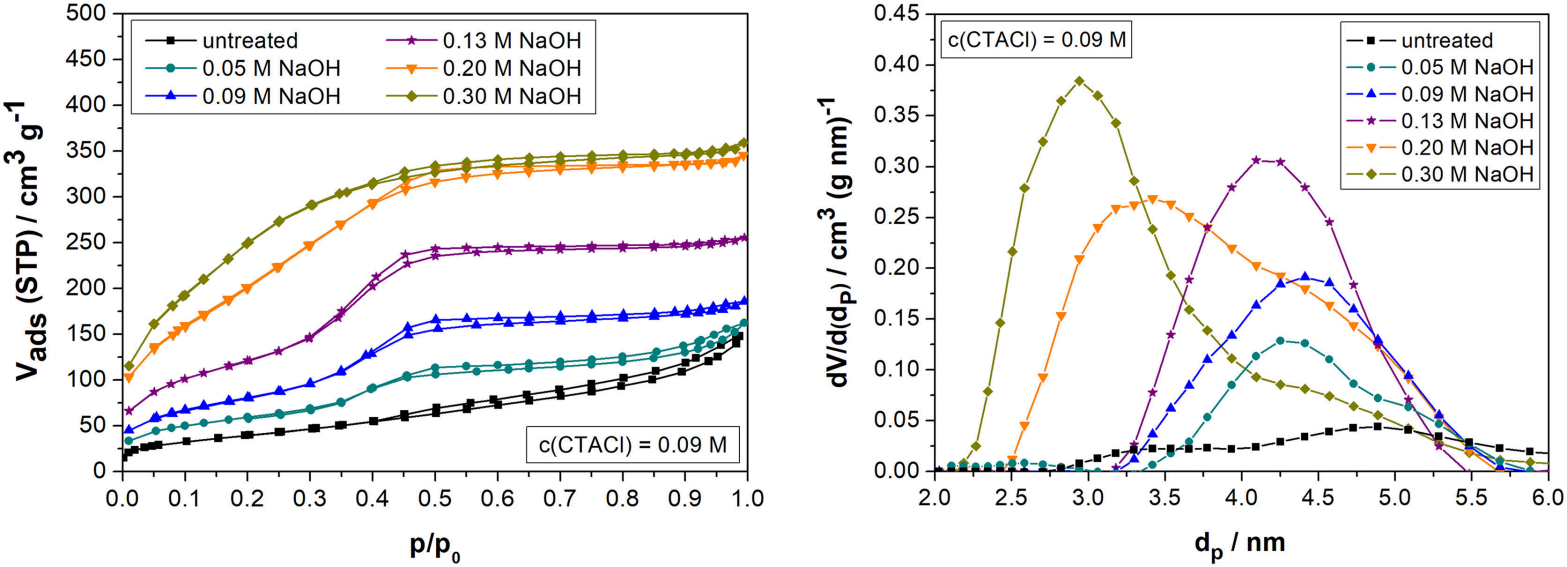
Nitrogen sorption isotherms (left) and pore width distributions (NLDFT method, right) of biogenic silica before and after partial transformation in the microwave for 10 min at 393 K with 0.09 M CTACl and 0.05, 0.09, 0.13, 0.20, or 0.30 M NaOH.

The starting material had a SiO_2_ content of 92.4 wt.% with 5.1 wt.% P_2_O_5_, 1.2 wt.% K_2_O, 0.5 wt.% MgO, and 0.3 wt.% CaO as the minor constituents and 0.5 wt.% other inorganic compounds, according to the XRF results. The biogenic silica exhibited an X-ray amorphous character (see Supplementary Material). Textural properties of the starting material determined by nitrogen sorption are given as a reference in Table 2 and Figures 1, 2, **5**. The isotherm can be characterized as type IV with H3 hysteresis; the biogenic silica shows a broad pore width distribution in the mesoporous range. The specific surface area according to BET was determined to be 147 m^2^ g^−1^ with a mesopore volume of 0.23 cm^3^ g^−1^. The isotherms do not reach a plateau near *p/p*_0_ = 1. That points out to larger meso- and macropores which cannot be completely filled with nitrogen. To determine the macropores, the technique of mercury intrusion would be necessary. However, since this method changes the texture of the fragile ashes, mercury intrusion was not carried out. The textural data of the starting material are similar to those from biogenic silica obtained in industrial scale (see Introduction). Hence, this material can serve as an appropriate model system.

**Table 2:** Textural properties (determined by nitrogen sorption) of biogenic silica before and after partial transformation in the drying oven for 24 h and in the microwave for 10 min at 393 K with either 0.09 M CTACl and 0.09 M NaOH (CTACl + NaOH), or 0.09 M CTABr and 0.09 M NaOH (CTABr + NaOH).

**Conditions**	***S_***BET***_*/m^**2**^ g^**−1**^**	***V_***P***_*/cm3 g^**−1**^**	***d_***P***_*/nm**
Untreated	147	0.23	5.1
CTACl + NaOH: Oven, 24 h	437	0.40	3.8
CTACl + NaOH: Microwave, 10 min	297	0.29	3.9
CTABr + NaOH: Microwave, 10 min	313	0.29	3.9

Partial pseudomorphic transformation of biogenic silica was carried out under the same conditions either in an oven for 24 h (convective heating) or in the microwave for 10 min using NaOH and Arquad® (CTACl) or CTABr solutions. The concentrations were chosen according to literature (Alyosef et al., 2014) and aimed for 50% transformation degree as this should approximately result in materials with half of the specific surface area and mesopore volume of fully transformed MCM-41 [i.e., *S*_*BET*_ ca. 500 m^2^ g^−1^and *V*_*P*_ ca. 0.5 cm^3^ g^−1^ for 50% transformation degree, depending on the surface area, pore volume, pore wall thickness, and pore width of the starting material (Uhlig et al., 2013)]. The textural parameters determined by nitrogen sorption are shown in Figure 1 and Table 2.

It can be deducted from Figure 1 (left) that the shape of the isotherm changes from type IV with a H3 hysteresis for the untreated biogenic silica to type IV with a small hysteresis and a steep uptake in the range of *p/p*_0_ = 0.35–0.40 for all partially transformed samples. This is due to the pores around 4 nm generated during the partial transformation (Figure 1, right).

When comparing the samples after reaction with Arquad® (CTACl) or CTABr in the microwave, the mesopore volume and specific surface are about comparable (Table 2, last two lines: 297 vs. 313 m^2^ g^−1^ and twice 0.29 cm^3^ g^−1^). In other words, the anion present in the surfactant has no significant influence on the textural parameters under these reaction conditions and the low-cost surfactant Arquad® is performing as good as the commonly used surfactant (CTABr) from previous literature (Galarneau et al., 2009).

When looking at the differences between oven and microwave synthesis using Arquad® and NaOH, the specific surface area and mesopore volume after microwave synthesis are only 30% smaller as compared to drying oven synthesis with conventional heating for 24 h (297 vs. 437 m^2^ g^−1^ and 0.29 vs. 0.40 cm^3^ g^−1^). However, the microwave synthesis is much more time efficient for partial transformation.

These changes in the textural parameters were also observed for other silica materials (HPLC silica gel and Aerosil® 90, see Supplementary Material), proving that the effects are independent of the starting material.

A higher reaction rate under microwave irradiation has been attributed to the higher heating rate (no thermal lag), a lower induction period as well as differences in crystal nucleation and growth during the synthesis of zeolites or related materials (Cundy and Zhao, 1998).

The thermal lag in conventional thermal heating is dependent on the reactor dimensions which can only be overcome by low volume-to-surface ratios. In contrast, the thermal lag for microwave heating is almost removed as the components are directly excited (Cundy and Zhao, 1998). In the case of dipolar compounds, the dipoles align to the oscillating microwave field which results in their rotation. The resulting friction between the components leads to an increase in the temperature (dielectric heating; Anwar et al., 2015). Ionic compounds experience an oscillation during microwave irradiation and thus create an electric current. Their collision with other atoms or molecules causes a heating effect (Kappe, 2004).

The induction period involves both dissolving and crystal growth. Other studies showed that the dissolution process of the silica precursor was faster when microwave heating was used (Wu and Bein, 1996; Slangen et al., 1997). Cundy and Zhao (1998) showed that the period of the initial growth of a crystal using nanometer scaled seed crystals is reduced to zero when microwave heating is applied. They assumed that the effects of microwave energization of hydroxylated species or water molecules are causing the specific energy dissipation which is responsible for the rapid activation of the seed crystals. This results in the differences in crystal nucleation and growth. In principle, these effects can be transferred to the formation of (pseudo crystalline) MCM-41.

The textural properties during partial pseudomorphic transformation can be varied by adjusting the Arquad® and NaOH concentration. The influence of the applied power (200–700 W) and reaction time (10–90 min) during microwave synthesis (biogenic silica, 393 K, 0.09 M NaOH, and 0.09 M CTACl) were also studied, but they did not show a remarkable effect on the textural properties.

### Control of the Textural Properties

#### Influence of the NaOH Concentration

The nitrogen sorption results after transformation in the microwave for a fixed surfactant concentration of 0.09 M CTACl and varying NaOH concentrations between 0.05 and 0.30 M are given in Figure 2 and Table 3.

**Table 3:** Textural properties (determined by nitrogen sorption) of biogenic silica before and after partial transformation in the microwave for 10 min at 393 K with 0.09 M CTACl and different NaOH concentrations.

***c*(NaOH)/M**	***S_***BET***_*/m^**2**^ g^**−1**^**	***V_***P***_*/cm3 g^**−1**^**	***d_***P***_*/nm**
Untreated	147	0.23	5.1
0.05	215	0.25	4.7
0.07	246	0.27	4.4
0.09	297	0.29	3.9
0.11	380	0.35	3.7
0.13	446	0.40	3.6
0.15	587	0.48	3.2
0.20	792	0.53	2.9
0.25	833	0.55	2.6
0.30	1,001	0.56	2.6

Within the investigated concentration range between 0.05 and 0.30 M NaOH, it can be deducted that the mesopore volume and specific surface area increase with the concentration of NaOH (Table 3). The highest specific surface area (1,001 m^2^ g^−1^) and mesopore volume (0.56 cm^3^ g^−1^) were reached by the sample using 0.30 M NaOH. The BET surface area increased to a factor of seven and the mesopore volumes detected by nitrogen physisorption was double as high as compared to the untreated material. This goes far beyond the initial target of a specific surface area of about 500 m^2^ g^−1^, since the desired degree of transformation was 50%, which thus theoretically (depending on the starting material and for a NaOH: SDA ratio of 1:1) provides a surface specific surface area of about 500 m^2^ g^−1^ and a mesopore volume of about 0.50 cm^3^ g^−1^. The values for the 0.30 M NaOH sample correspond to literature values for biogenic silica after 1–2 days of pseudomorphic transformation using convective heating (*S*_*BET*_ = 718–918 m^2^ g^−1^ and *V*_*p*_ = 0.51–0.64 cm^3^ g^−1^; Alyosef et al., 2014). Voegtlin et al. (1997) found that a higher pH value during transformation leads to a smaller degree of polycondensation. This results in an increase of pore shrinking during calcination and leaves smaller pores as compared to the typical 4 nm CTA^+^ templated MCM-41 pores which were formed for the samples up to 0.15 M NaOH. This effect can be clearly observed in the changes of the pore width distribution (Figure 2, right).

The isotherms (Figure 2, left) reveal that the shape of the isotherms for the samples up to 0.15 M NaOH changes from IV(b) to a type I(b) isotherm for the samples with a higher NaOH concentration. This isotherm shape is characteristic for materials with a broad pore width distribution including large micropores and/or narrow mesopores of around 2.5 nm or less (Thommes et al., 2015), which is reflected in the pore width distribution (Figure 2, right). The hysteresis (H1) points out that parts of the larger mesopores of the starting material are only accessible through the smaller mesopores. The steep ascent at *p/p*_0_ = 0.35–0.40 typical for MCM-41 materials cannot be observed for the samples synthesized with more than 0.15 M NaOH. This leads to the assumption that the formed materials do not contain any characteristics of an MCM-41 phase. That means, the reaction took place without reprecipitation into a detectable ordered mesoporous structure. This might be due to a deficit amount of surfactant as compared to the high NaOH concentration (**Figure 7**). Instead of this, the silica material might have been deposited and additional small mesopores of 2–3 nm were formed (Figure 2, right). The presence of these small mesopores could be the reason for the high specific surface area of more than 800 m^2^ g^−1^ for the samples obtained with a NaOH concentration between 0.20 and 0.30 M. However, the high specific surface areas of these materials are remarkable, but were not the main goal of this study.

**Figure 3:**
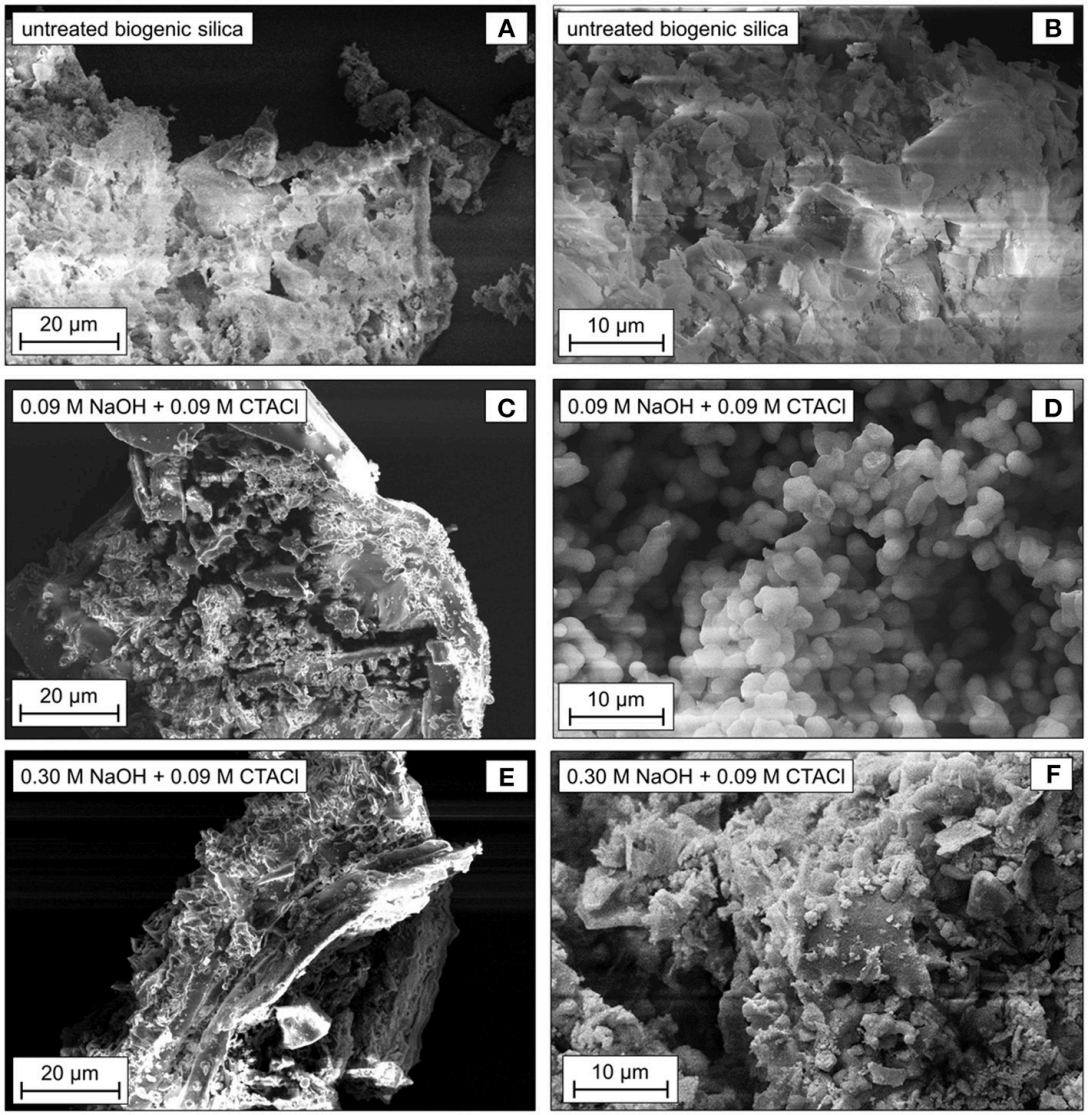
SEM images of untreated biogenic silica **(A,B)** and after partial transformation in the microwave for 10 min at 393 K with 0.09 M **(C,D)** or 0.30 M NaOH **(E,F)** and 0.09 M CTACl.

**Figure 4:**
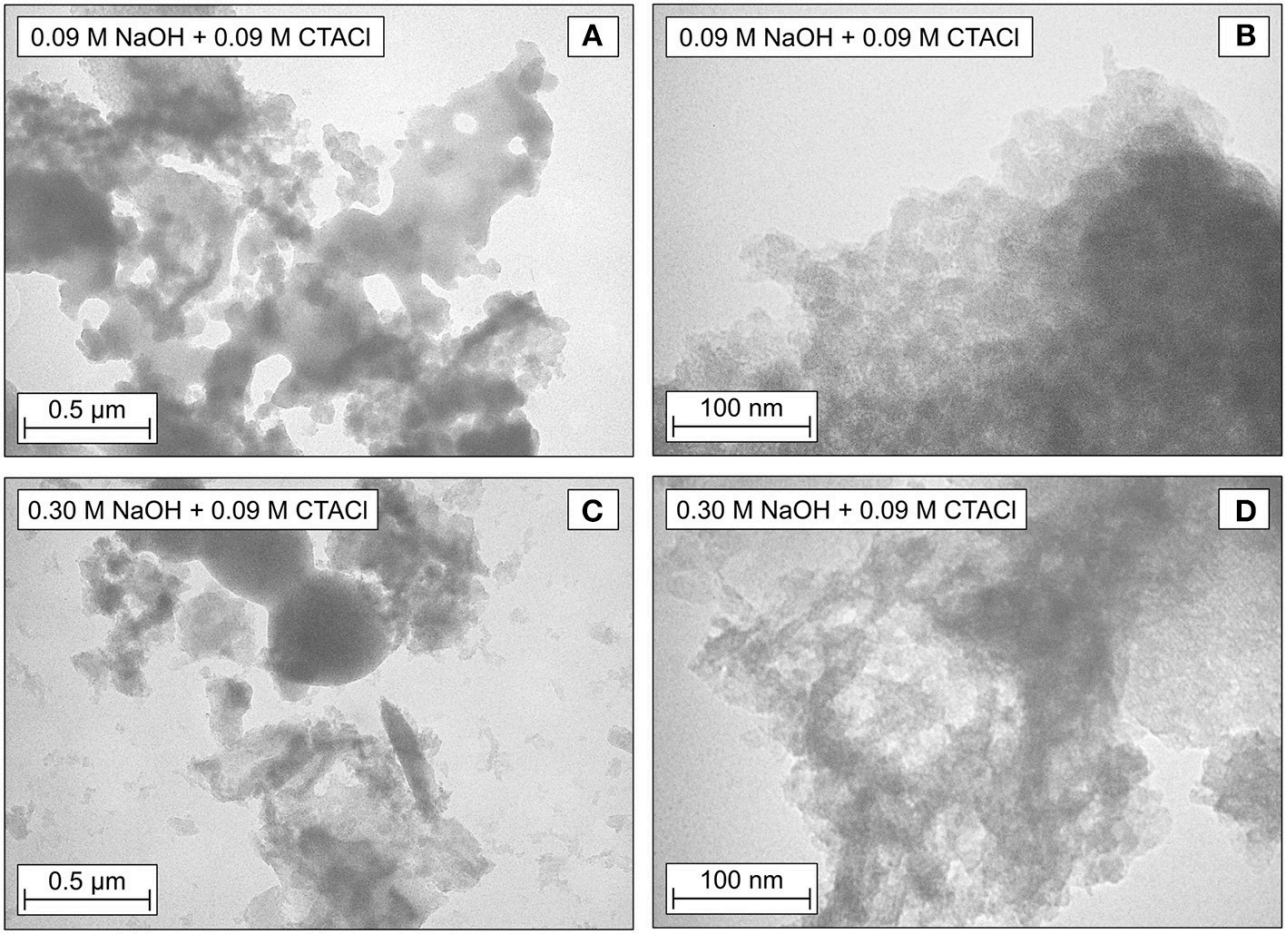
TEM images of materials after partial transformation in the microwave for 10 min at 393 K with 0.09 M NaOH and 0.09 M CTACl **(A,B)** as well as 0.30 M NaOH and 0.09 M CTACl **(C,D)**.

To study the structural properties of the obtained materials, selected samples were investigated by SEM and TEM (Figures 3, 4).

The SEM images reveal that the untreated biogenic silica exhibits fragments of the initial husk structure (Figures 3A,B). After partial transformation using 0.09 M NaOH, the husk morphology was retained (Figure 3C). Three-dimensionally connected, round particles can be observed for the sample after transformation using 0.09 M NaOH (Figure 3D). The size of the small spherical particles was determined to be around 2–3 μm. They resemble the MCM-41 domains observed by Uhlig et al. (2018) and Alyosef et al. (2014), but are larger in size. The sample after transformation with 0.30 M NaOH also contains husk backbone fragments (Figure 3E). In contrast to the sample using 0.09 M NaOH, no homogenously shaped particles can be observed (Figure 3F). These findings clearly confirm a structural change of the partially transformed samples as compared to the starting material (Figures 3D,F as compared to Figure 3B). However, the outer morphology of the biogenic silica and the macropores were preserved (Figures 3A,C,E). To this end, the reaction can be designated as pseudomorphic transformation (Galarneau et al., 2006; Inayat et al., 2013; Uhlig et al., 2013).

The TEM pictures (Figure 4) show agglomeration as typical for biogenic silica nanoparticles. As compared to the TEM of untreated biogenic silica (Schneider et al., 2018), a small indication of chamfering can be observed (Figures 4A,C), which might be due to the alkaline conditions in the reaction mixture. However, none of the samples exhibits a clear indication of an MCM-41 typical hexagonally ordered pore system as described in the TEM studies in literature (Beck et al., 1992; Kresge et al., 1992). Additional small-angle X-Ray scattering (SAXS) patterns show a resemblance to silica gel and confirm the poor long-range order of the synthesized materials (see Supplementary Material).

The mechanism of the transformation of biogenic silica is assumed to resemble silica gel as the small mesopores in biogenic silica are similar to amorphous silica gel. Previous studies of our group showed that the silica network of silica gel is transformed from the outside to the inside (Einicke et al., 2013a). The space inside the pore system of the starting material particles is limited and the pore structure/arrangement is disordered. This given structure remains largely unchanged during the transformation. Thus, only a low degree of long-range order can be achieved. The formation of small MCM-41 domains was proven by PFG-NMR studies (Einicke et al., 2013a). Martin et al. (2002) also carried out studies on the transformation of silica gel to MCM-41. The authors assumed that the silica/surfactant ratio inside the grains of the silica gel starting material is higher than the total system composition in the early stage of the transformation. This generates a micelle-templated phase with thick walls of silica and a poor long-range order. Our previous study investigated the transformation of biogenic silica to MCM-41 (Alyosef et al., 2014). As proven by microscopic studies, the MCM-41 material was formed on the outer surface of the biogenic silica, which gives evidence to the assumption that the silica is dissolved from the outside of the particles to the inside.

These results can be transferred to the present study. The short reaction time applied in this work leads to the assumption that only the first stage of the transformation was reached. The formation of the MCM-41 on the outer surface of the parent biogenic silica material explains the formation for the particles as depicted in the SEM images (Figure 3D). As the MCM-41 is not formed in the volume-phase, the TEM images are not able to represent the typical MCM-41 hexagonal structures. The SAXS patterns prove the missing structural order of the synthesized materials.

Since a NaOH concentration of 0.13 M (with a fixed concentration of 0.09 M CTACl) resulted in textural properties in the range of the initial target, the NaOH concentration of 0.13 M was kept constant for the subsequent investigations. For economic and ecologic reasons, a lowering of the tenside concentration is favorable. The according results are reported in the next section.

#### Influence of the CTACl Concentration

**Figure 5:**
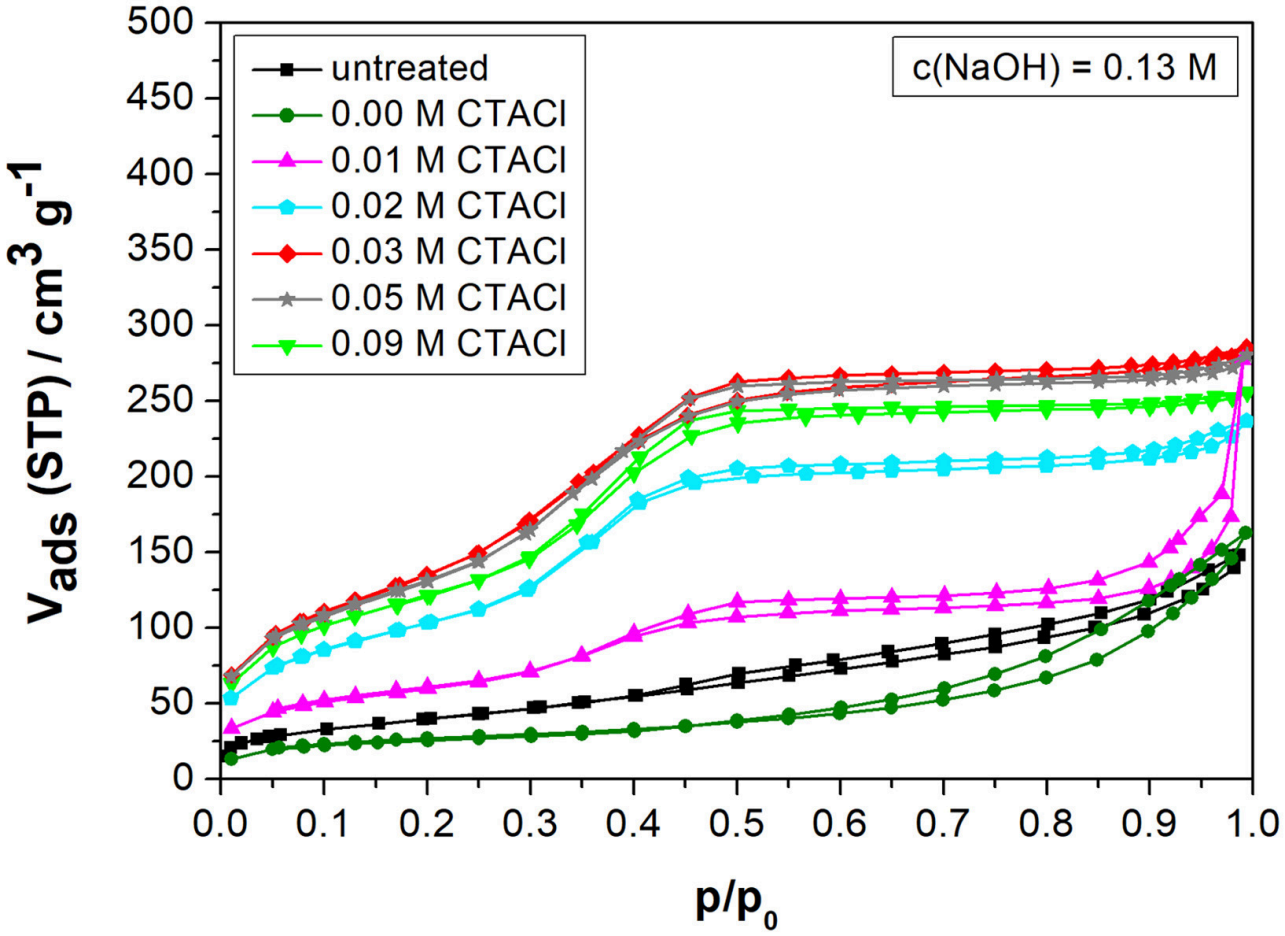
Nitrogen sorption isotherms of biogenic silica before and after partial transformation in the microwave for 10 min at 393 K with 0.13 M NaOH and 0.00, 0.01, 0.02, 0.03, 0.05, or 0.09 M CTACl.

The nitrogen sorption results after partial transformation of biogenic silica in the microwave for a fixed NaOH concentration of 0.13 M and varying CTACl concentrations up to 0.09 M are given in Figure 5 and Table 4.

**Table 4:** Textural properties (determined by nitrogen sorption) of biogenic silica before and after partial transformation in the microwave for 10 min at 393 K with 0.13 M NaOH and different surfactant concentrations.

***c*(CTACl)/M**	***S_***BET***_*/m^**2**^ g^**−1**^**	***V_***P***_*/cm3 g^**−1**^**	***d_***P***_*/nm**
Untreated	147	0.23	5.1
0.00	97	0.25	10.7
0.01	218	0.43	7.9
0.02	380	0.37	3.9
0.03	505	0.44	3.5
0.05	487	0.43	3.5
0.07	449	0.38	3.4
0.09	446	0.40	3.6

Applying only NaOH (without CTACl) leads to a pore widening and therefore to a decrease in the specific surface area. The isotherm of this specimen reveals that no MCM-41 phase is formed as the MCM-41 typical steep ascent at *p/p*_0_ = 0.35–0.40 cannot be observed. In the range of 0.01 to 0.03 M CTACl, the isotherms of type IV prove the partial presence of MCM-41 like phases. The small hysteresis (H1) implies that parts of the larger mesopores of the starting material are only accessible through smaller mesopores. The specific surface area increases with a higher concentration of surfactant between 0.01 and 0.03 M CTACl, whereas the mesopore volume does not change significantly. According to literature, the critical micelle concentration for CTACl is at 0.5 wt.% (Cai et al., 1999) which falls in the concentration range of 0.01–0.02 M in this study. Hence, the surfactant is not forming a sufficient number of structure-directing micelles in this range, which explains the observed effects. Any further rise in the surfactant concentration above 0.03 M has no significant effect on the textural properties (Table 4). That means, the surfactant consumption can be reduced from 0.09 to 0.03 M to achieve almost similar material characteristics in the range of the initial target values as when using the conventional convective heating method for 24 h, hence saving costs for the process.

**Figure 6:**
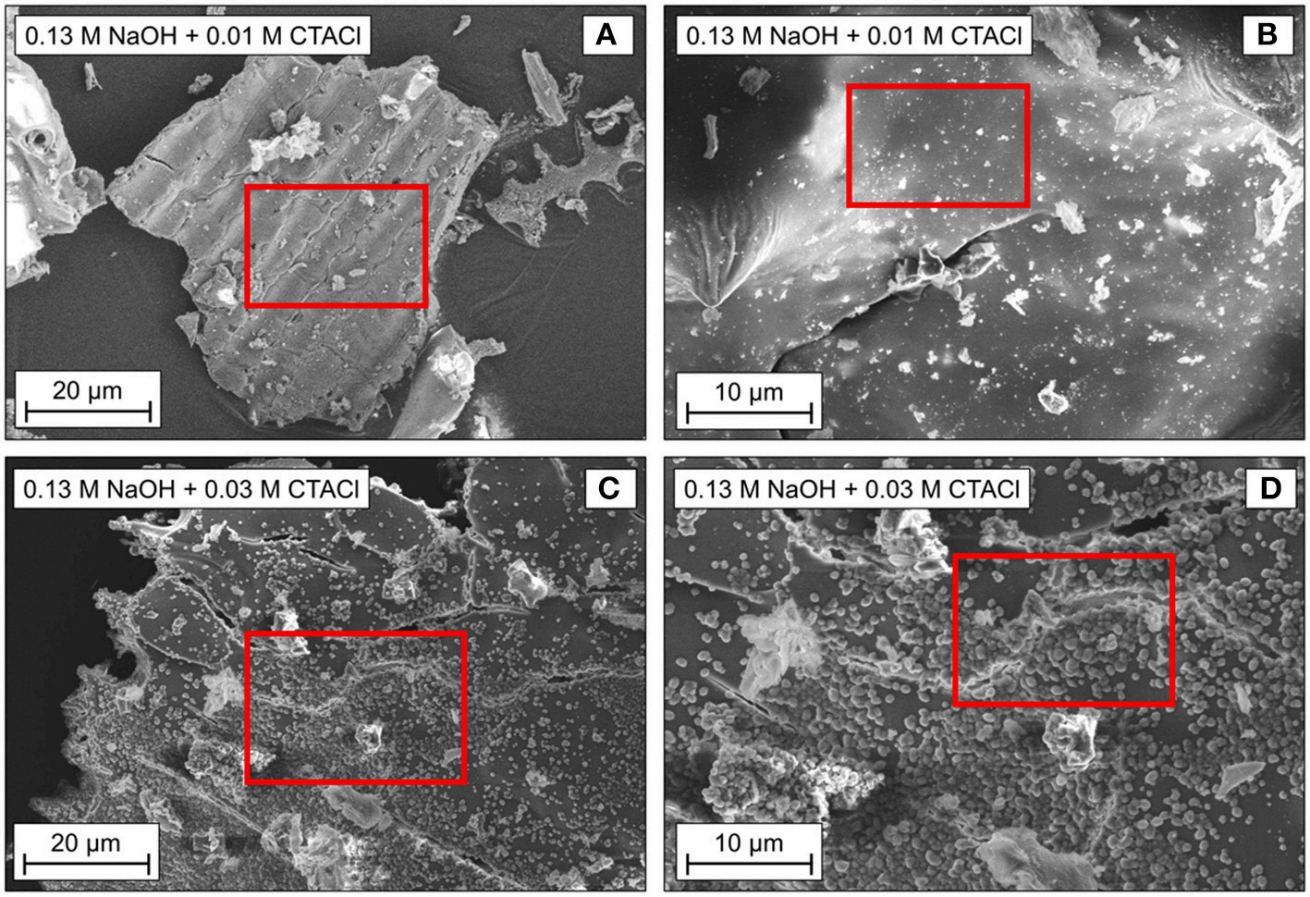
SEM images of the samples obtained after partial transformation in the microwave for 10 min at 393 K with 0.01 M **(A,B)** or 0.03 M **(C,D)** CTACl and 0.13 M NaOH.

The structures of the samples after treatment with 0.13 M NaOH and 0.01 or 0.03 M CTACl were studied by SEM (Figure 6). Both show the backbone of the initial spelt husk ash, proving the preservation of the macroscopic shape during partial pseudomorphic transformation. However, the specimen after treatment with 0.01 M CTACl and 0.13 M NaOH (Figures 6A,B) exhibits a relatively smooth outer surface. In contrast to that, the outer surface of sample treated with 0.03 M CTACl and 0.13 M NaOH (Figures 6C,D) contains small particles similar to those in the sample 0.09 M NaOH and 0.09 M CTACl (Figures 3C,D). These particles might have been formed during partial transformation and might explain the better textural properties of this sample as compared to the sample using 0.01 M CTACl, where no significant changes in the structure were observed (Table 4). As discussed in the previous section, the formation of the particles on the outer surface after transformation is due to the pore structure of the starting material and the associated mechanism.

#### Summary of the Influence of the Ratio of NaOH and CTACl

**Figure 7:**
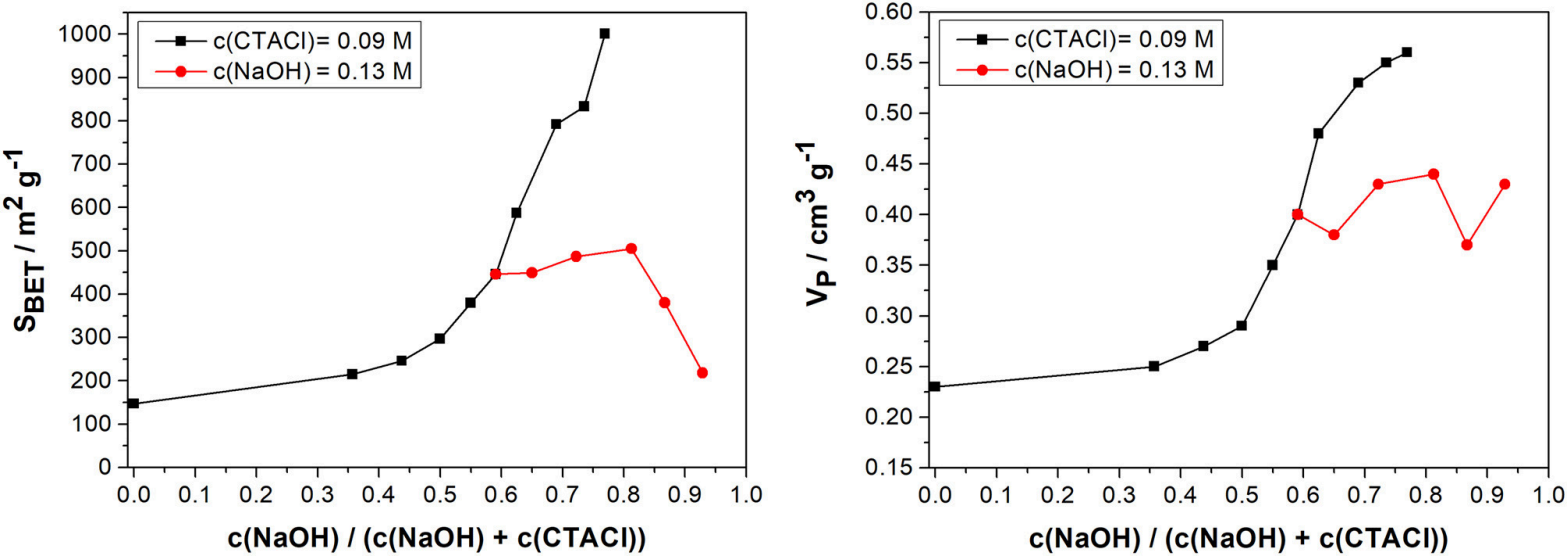
Specific surface area (*S*_*BET*_, left) and mesopore volume (*V*_*P*_, right) [determined by nitrogen sorption) of the transformed biogenic silica in dependence of the ratio *c(NaOH)/(c(NaOH)* + *c(CTACl)*] in the applied transformation solution for either a fixed CTACl concentration (squared symbols) or NaOH concentration (round symbols).

Figure 7 provides a summary of the obtained textural data in dependence of the ratio of the applied NaOH concentration to the sum of the NaOH and CTACl concentration in the transformation solution for either a fixed NaOH or CTACl concentration. According to literature, the ratio for obtaining fully transformed MCM-41 from biogenic silica using convective heating is around 0.5 (Alyosef et al., 2014).

Between a ratio of 0 and 0.6 during the variation of the NaOH concentration, the specific surface area and the mesopore volume increase with a higher ratio (as discussed in Influence of the NaOH Concentration). This trend was already observed by Uhlig et al. (2013) for the transformation of porous glasses using convective heating. However, above a ratio of 0.6, the different ratios were adjusted with a fixed NaOH concentration (0.13 M, *c(CTACl)* = 0.01–0.09 M) or a fixed CTACl concentration (0.09 M, *c(NaOH)* = 0.15–0.30 M). In this range, the achievable specific surface areas and mesopore volumes do not only depend on the ratio, but also on the absolute concentration of NaOH and CTACl in the transformation solution under the applied conditions. One explanation would be that the concentration of 0.13 M NaOH during the variation of the surfactant concentration is not sufficient to dissolve enough SiO_2_ under the applied synthesis conditions (round symbols in Figure 7). Another possibility would be that the dissolution rate during the microwave synthesis of 10 min is too low. This would explain the stagnation in the textural properties during the decrease of the CTACl concentration (ratio: 0.6–0.8). However, a surfactant concentration below 0.03 M (ratio > 0.8) is not forming a sufficient number of structure-directing micelles which might cause the decrease in the textural properties. The results lead to the assumption that the partial transformation under the applied conditions is kinetically controlled, which is in accordance with literature (Cai et al., 1999).

In the case of increasing the NaOH concentration, the surfactant concentration (0.09 M) enables the formation MCM-41 like structures until 0.15 M NaOH (ratio of 0.6, squared symbols in Figure 7). With a NaOH concentration of higher than that, the silica will get redistributed under the formation of mesopores smaller than the typical 4 nm MCM-41 pores. This leads to the steep increase in the specific surface area and the mesopore volume.

Regarding the initial target range for the specific surface area and mesopore volume and considering economic reasons (low tenside concentration), the batch using 0.13 M NaOH and 0.03 M CTACl is the most favorable approach within this study.

### Assessment of the Economic Advantages

Microwave-assisted synthesis may be considered as a time- and cost-efficient way to conduct reactions. As compared to conventional convective heating, the reaction time in this study could be reduced by more than 99% from 24 h to 10 min. The material after synthesis using convective heating (24 h, 0.09 M CTACl and 0.09 M NaOH) showed a specific surface area of 437 m^2^ g^−1^ and a mesopore volume of 0.40 cm^3^ g^−1^ (Table 2). About equal textural properties (*S*_*BET*_ = 446 m^2^ g^−1^ and *V*_*P*_ = 0.40 cm^3^ g^−1^) were obtained after microwave synthesis for 10 min using 0.09 M CTACl and 0.13 M NaOH. Hence, only a small excess of low-cost NaOH must be applied during rapid microwave synthesis to achieve the same textural parameters as with convective heating over 24 h.

According to the manuals of the devices, the power consumption of the microwave is roughly double the amount of the oven used in this study. That means, the energy efficiency of the microwave is less than in convective heating (Nüchter et al., 2003, 2004). However, this must be put into the perspective of the over hundred-fold higher “sample” throughput in the microwave due to the short reaction time (10 min vs. 24 h).

In addition, a cost calculation was carried out for Germany (2018). The price of the surfactant solution Arquad® 16-29 is by 76–99% lower than a comparable solution of CTACl from other suppliers, and 90–99% less than for the conventionally used CTABr (prices obtained from Julius Hoesch GmbH & Co. KG, Sigma-Aldrich and compiled from https://de.vwr.com/store/ as of April 2018). As already discussed, the tenside concentration can be lowered by more than 60% as compared to the standard procedure without a remarkable effect on the textural properties.

For future work, the removal process of the tenside could be accelerated by using ion exchange, microwave digestion plasma process, and sonication to make the overall procedure even faster (Gérardin et al., 2013). It would be also interesting to use biogenic silica obtained from industrial scale. In addition, it is worth considering to conduct the partial transformation as a continuous reaction in a microwave flow reactor (Estel et al., 2017) to overcome the drawback of the small penetration depth of the microwaves and to scale up the synthesis.

## Conclusions

The aim of this study was to introduce a cost and time efficient method to specifically increase the mesopore volume and specific surface area of biogenic silica to ~0.40–0.50 cm^3^ g^−1^ and ~500 m^2^ g^−1^ by partial pseudomorphic transformation in a microwave reactor using a low-cost surfactant.

The textural parameters during partial transformation can be controlled by the NaOH concentration in the reaction solution, enabling the synthesis of tailor-made materials with specific surface areas ranging from 215 to 1,000 m^2^ g^−1^ and mesopore volumes between 0.25 and 0.56 cm^3^ g^−1^. An increase in the NaOH concentration for 0.09 M CTACl resulted in a raise of the specific surface area and the mesopore volume. It was shown that a NaOH concentration of > 0.15 M leads to a disproportionally increase of the specific surface area as compared to the mesopore volume. This raise in the specific surface area might be due to the formation of additional small mesopores of 2–3 nm in size. However, these high surface areas are far beyond the initial target of a specific surface area of ca. 500 m^2^ g^−1^ and the materials produced with a NaOH concentration of higher than 0.15 M are highly disordered without any characteristics of an MCM-41 phase. For a constant NaOH concentration of 0.13 M, the specific mesopore volume and surface area increased with the concentration of tenside up to 0.03 M. Any further rise did not influence the textural properties which lead to the assumption that the partial transformation under the applied conditions is kinetically controlled. However, the surfactant consumption can be reduced from 0.09 to 0.03 M, saving costs for the preparation process. The batch using 0.13 M NaOH and 0.03 M CTACl was found to be the most economic and ecologic approach to produce an MCM-41 like material in the range of the initial targets (*S*_*BET*_ = 505 m^2^ g^−1^ and *V*_*P*_ = 0.44 cm^3^ g^−1^).

Materials with a specific surface of > 500 m^2^ g^−1^ were additionally prepared as products beyond these target values. The highest textural properties were obtained after reaction of biogenic silica with 0.30 M NaOH and 0.09 M CTACl (*V*_*P*_ = 0.56 cm^3^ g^−1^ and *S*_*BET*_ = 1001 m^2^ g^−1^. These values are superior or comparable to the adsorbents reported in the introduction (Table 1) with the advantageous feature that the material in this study was synthesized in a cheaper and faster procedure (direct one-pot synthesis as compared to the synthesis of sodium silicate solutions which are then used for templated mesoporous silica synthesis).

Scanning electron microscopic (SEM) and TEM studies revealed that the reaction affects the structure of the biogenic silica. However, the macroscopic backbone structure and size of the biogenic starting material were retained during transformation, indicating the pseudomorphic character.

The presented microwave-assisted route using the low-cost detergent solution Arquad® 16-29 (containing 29 wt.% CTACl) reduces the preparation costs as compared to the route using CTABr in an oven significantly: the reaction time was decreased by 99% and as shown in an example for Germany, the costs for the used chemicals were reduced by 76–99%.

## Author Contributions

DS, RK, and W-DE conceived and designed the experiments. RK synthesized the materials and analyzed the raw data. W-DE carried out the nitrogen physisorption measurements. DS wrote the manuscript. All authors contributed to the evaluation of the results and to the manuscript revision.

### Conflict of Interest Statement

The authors declare that the research was conducted in the absence of any commercial or financial relationships that could be construed as a potential conflict of interest.

## References

[B1] Alyosef H. A. Eilert A. Welscher J. Ibrahim S. S. Denecke R. Schwieger W. (2013). Characterization of biogenic silica generated by thermo chemical treatment of rice husk. Part. Sci. Technol. 31, 524–532. 10.1080/02726351.2013.782931

[B2] Alyosef H. A. Schneider D. Wassersleben S. Roggendorf H. Weiß M. Eilert A. (2015). Meso/Macroporous silica from miscanthus, cereal remnant pellets and wheat straw. ACS Sustainable Chem. Eng. 3, 2012–2021. 10.1021/acssuschemeng.5b00275

[B3] Alyosef H. A. Uhlig H. Münster T. Kloess G. Einicke W.-D. Gläser R. (2014). Biogenic silica from rice husk ash - sustainable sources for the synthesis of value added silica. Chem. Eng. Trans. 37, 667–672. 10.3303/CET1437112

[B4] Anwar J. Shafique U. Zaman W.-U. Rehman R. Salman M. Dar A. (2015). Microwave chemistry: effect of ions on dielectric heating in microwave ovens. Arab. J. Chem. 8, 100–104. 10.1016/j.arabjc.2011.01.014

[B5] Arami-Niya A. Wan Daud W. M. A. Mjalli F. S. Abnisa F. Shafeeyan M. S. (2012). Production of microporous palm shell based activated carbon for methane adsorption: modeling and optimization using response surface methodology. Chem. Eng. Res. Des. 90, 776–784. 10.1016/j.cherd.2011.10.001

[B6] Beck J. S. Vartuli J. C. Roth W. J. Leonowicz M. E. Kresge C. T. Schmitt K. D. (1992). A new family of mesoporous molecular sieves prepared with liquid crystal templates. J. Am. Chem. Soc. 114, 10834–10843. 10.1021/ja00053a020

[B7] Bhagiyalakshmi M. Yun L. J. Anuradha R. Jang H. T. (2010). Utilization of rice husk ash as silica source for the synthesis of mesoporous silicas and their application to CO2 adsorption through TREN/TEPA grafting. J. Hazard. Mater. 175, 928–938. 10.1016/j.jhazmat.2009.10.097 19939554

[B8] Brunauer S. Emmett P. H. Teller E. (1938). Adsorption of gases in multimolecular layers. J. Am. Chem. Soc. 60, 309–319. 10.1021/ja01269a023

[B9] Budinova T. Savova D. Tsyntsarski B. Ania C. O. Cabal B. Parra J. B. (2009). Biomass waste-derived activated carbon for the removal of arsenic and manganese ions from aqueous solutions. Appl. Surf. Sci. 255, 4650–4657. 10.1016/j.apsusc.2008.12.013

[B10] Cai Q. Lin W. Y. Xiao F. S. Pang W. Q. Chen X. H. Zou B. S. (1999). The preparation of highly ordered MCM-41 with extremely low surfactant concentration. Micropor. Mesopor. Mat. 32, 1–15. 10.1016/S1387-1811(99)00082-7

[B11] Chandrasekar G. Son W.-J. Ahn W.-S. (2009). Synthesis of mesoporous materials SBA-15 and CMK-3 from fly ash and their application for CO_2_ adsorption. J. Porous Mater. 16, 545–551. 10.1007/s10934-008-9231-x

[B12] Chandrasekar G. You K.-S. Ahn J.-W. Ahn W.-S. (2008). Synthesis of hexagonal and cubic mesoporous silica using power plant bottom ash. Micropor. Mesopor. Mat. 111, 455–462. 10.1016/j.micromeso.2007.08.019

[B13] Chareonpanich M. Namto T. Kongkachuichay P. Limtrakul J. (2004). Synthesis of ZSM-5 zeolite from lignite fly ash and rice husk ash. Fuel Process. Technol. 85, 1623–1634. 10.1016/j.fuproc.2003.10.026

[B14] Chen C.-Y. Burkett S. L. Li H.-X. Davis M. E. (1993). Studies on mesoporous materials II. Synthesis mechanism of MCM-41. Micropor. Mater. 2, 27–34. 10.1016/0927-6513(93)80059-4

[B15] Cheng Y. Lu M. Li J. Su X. Pan S. Jiao C.. (2012). Synthesis of MCM-22 zeolite using rice husk as a silica source under varying-temperature conditions. J. Colloid Interface Sci. 369, 388–394. 10.1016/j.jcis.2011.12.024 22226498

[B16] Chiarakorn S. Areerob T. Grisdanurak N. (2007). Influence of functional silanes on hydrophobicity of MCM-41 synthesized from rice husk. Sci. Technol. Adv. Mater. 8, 110–115. 10.1016/j.stam.2006.11.011

[B17] Cundy C. S. Zhao J. P. (1998). Remarkable synergy between microwave heating and the addition of seed crystals in zeolite synthesis—a suggestion verified. Chem. Commun., 1465–1466. 10.1039/a803324b

[B18] Dasgupta J. Kumar A. Mandal D. D. Mandal T. Datta S. (2015). Removal of phenol from aqueous solutions using adsorbents derived from low-cost agro-residues. Desalin. Water Treat. 57, 14188–14212. 10.1080/19443994.2015.1061455

[B19] Daud W. M. A. W. Ali W. S. W. (2004). Comparison on pore development of activated carbon produced from palm shell and coconut shell. Bioresour. Technol. 93, 63–69. 10.1016/j.biortech.2003.09.015 14987722

[B20] Dodson J. R. Cooper E. C. Hunt A. J. Matharu A. Cole J. Minihan A. (2013). Alkali silicates and structured mesoporous silicas from biomass power station wastes: the emergence of bio-MCMs. Green Chem. 15, 1203–1210. 10.1039/c3gc40324f

[B21] Dutta S. Bhattacharyya A. Ganguly A. Gupta S. Basu S. (2011). Application of response surface methodology for preparation of low-cost adsorbent from citrus fruit peel and for removal of methylene blue. Desalination 275, 26–36. 10.1016/j.desal.2011.02.057

[B22] Einicke W.-D. Enke D. Dvoyashkin M. Valiullin R. Gläser R. (2013a). The mechanism of pseudomorphic transformation of spherical silica gel into MCM-41 studied by PFG NMR diffusometry. Materials 6, 3688–3709. 10.3390/ma6093688 28788300 PMC5452651

[B23] Einicke W.-D. Uhlig H. Enke D. Gläser R. Reichenbach C. Ebbinghaus S. G. (2013b). Synthesis of hierarchical micro/mesoporous Y-zeolites by pseudomorphic transformation. Colloids Surf. A Physicochem. Eng. Asp. 437, 108–112. 10.1016/j.colsurfa.2012.12.024

[B24] Endud S. Wong K.-L. (2007). Mesoporous silica MCM-48 molecular sieve modified with SnCl_2_ in alkaline medium for selective oxidation of alcohol. Micropor. Mesopor. Mat. 101, 256–263. 10.1016/j.micromeso.2006.12.029

[B25] Estel L. Poux M. Benamara N. Polaert I. (2017). Continuous flow-microwave reactor: where are we? Chem. Eng. Process. 113, 56–64. 10.1016/j.cep.2016.09.022

[B26] Ferch H. (1976). Pulverförmige amorphe synthetische kieselsäure-produkte herstellung und charakterisierung. CIT 48, 922–933. 10.1002/cite.330481104

[B27] Firouzi A. Kumar D. Bull L. Besier T. Sieger P. Huo Q.. (1995). Cooperative organization of inorganic-surfactant and biomimetic assemblies. Science 267, 1138–1143. 10.1126/science.7855591 7855591

[B28] Foo K. Y. Hameed B. H. (2009). Utilization of rice husk ash as novel adsorbent: A judicious recycling of the colloidal agricultural waste. Adv. Colloid Interface Sci. 152, 39–47. 10.1016/j.cis.2009.09.005 19836724

[B29] Galarneau A. Calin N. Iapichella J. Barrande M. Denoyel R. Coasne B. (2009). Optimization of the properties of macroporous chromatography silica supports through surface roughness control. Chem. Mater. 21, 1884–1892. 10.1021/cm803456t

[B30] Galarneau A. Iapichella J. Bonhomme K. Di Renzo F. Kooyman P. Terasaki O. (2006). Controlling the morphology of mesostructured silicas by pseudomorphic transformation: A route towards applications. Adv. Funct. Mater. 16, 1657–1667. 10.1002/adfm.200500825

[B31] Gérardin C. Reboul J. Bonne M. Lebeau B. (2013). Ecodesign of ordered mesoporous silica materials. Chem. Soc. Rev. 42, 4217–4255. 10.1039/c3cs35451b 23407854

[B32] Ghorbani F. Younesi H. Mehraban Z. Çelik M. S. Ghoreyshi A. A. Anbia M. (2013). Preparation and characterization of highly pure silica from sedge as agricultural waste and its utilization in the synthesis of mesoporous silica MCM-41. J. Taiwan. Inst. Chem. Eng. 44, 821–828. 10.1016/j.jtice.2013.01.019

[B33] Gonsalvesh L. Yperman J. Carleer R. Mench M. Herzig R. Vangronsveld J. (2016). Valorisation of heavy metals enriched tobacco biomass through slow pyrolysis and steam activation. J. Chem. Technol. Biotechnol. 91, 1585–1595. 10.1002/jctb.4889

[B34] Hesas R. H. Arami-Niya A. Daud W. M. A. W. Sahu J. N. (2013). Preparation and characterization of activated carbon from apple waste by microwave-assisted phosphoric acid activation: Application in methylene blue adsorption. BioResources 8, 2950–2966. 10.15376/biores.8.2.2950-2966

[B35] Hui K. S. Chao C. Y. H. (2006). Synthesis of MCM-41 from coal fly ash by a green approach: Influence of synthesis pH. J. Hazard. Mater. 137, 1135–1148. 10.1016/j.jhazmat.2006.03.050 16647813

[B36] Inayat A. Reinhardt B. Uhlig H. Einicke W.-D. Enke D. (2013). Silica monoliths with hierarchical porosity obtained from porous glasses. Chem. Soc. Rev. 42, 3753–3764. 10.1039/c2cs35304k 23081802

[B37] Kappe C. O. (2004). Controlled microwave heating in modern organic synthesis. Angew. Chem. Int. Edit. 43, 6250–6284. 10.1002/anie.200400655 15558676

[B38] Kordatos K. Gavela S. Ntziouni A. Pistiolas K. N. Kyritsi A. Kasselouri-Rigopoulou V. (2008). Synthesis of highly siliceous ZSM-5 zeolite using silica from rice husk ash. Micropor. Mesopor. Mat. 115, 189–196. 10.1016/j.micromeso.2007.12.032

[B39] Kresge C. T. Leonowicz M. E. Roth W. J. Vartuli J. C. Beck J. S. (1992). Ordered mesoporous molecular sieves synthesized by a liquid-crystal template mechanism. Nature 359, 710 10.1038/359710a0

[B40] Kumar P. Mal N. Oumi Y. Yamana K. Sano T. (2001). Mesoporous materials prepared using coal fly ash as the silicon and aluminium source. J. Mater. Chem. 11, 3285–3290. 10.1039/B104810B

[B41] Lakshmi U. R. Srivastava V. C. Mall I. D. Lataye D. H. (2009). Rice husk ash as an effective adsorbent: Evaluation of adsorptive characteristics for Indigo Carmine dye. J. Environ. Manag. 90, 710–720. 10.1016/j.jenvman.2008.01.002 18289771

[B42] Lataye D. H. Mishra I. M. Mall I. D. (2008). Pyridine sorption from aqueous solution by rice husk ash (RHA) and granular activated carbon (GAC): Parametric, kinetic, equilibrium and thermodynamic aspects. J. Hazard. Mater. 154, 858–870. 10.1016/j.jhazmat.2007.10.111 18082952

[B43] Li C.-,c. Qiao X.-,c. Yu J.-,g. (2016). Large surface area MCM-41 prepared from acid leaching residue of coal gasification slag. Mater. Lett. 167, 246–249. 10.1016/j.matlet.2015.12.125

[B44] Martin T. Galarneau A. Di Renzo F. Fajula F. Plee D. (2002). Morphological control of MCM-41 by pseudomorphic synthesis. Angew. Chem. Int. Ed. 41, 2590–2592. 10.1002/1521-3773(20020715)41:14<2590:AID-ANIE2590>3.0.CO;2-3 12203544

[B45] Misran H. Singh R. Begum S. Yarmo M. A. (2007). Processing of mesoporous silica materials (MCM-41) from coal fly ash. J. Mater. Process. Technol. 186, 8–13. 10.1016/j.jmatprotec.2006.10.032

[B46] Mohan D. Singh K. P. Singh V. K. (2008). Wastewater treatment using low cost activated carbons derived from agricultural byproducts–a case study. J. Hazard. Mater. 152, 1045–1053. 10.1016/j.jhazmat.2007.07.079 17951000

[B47] Monnier A. Schüth F. Huo Q. Kumar D. Margolese D. Maxwell R. S.. (1993). Cooperative formation of inorganic-organic interfaces in the synthesis of silicate mesostructures. Science 261, 1299–1303. 10.1126/science.261.5126.1299 17731857

[B48] Nüchter M. Müller U. Ondruschka B. Tied A. Lautenschläger W. (2003). Microwave-assisted chemical reactions. Chem. Eng. Technol. 26, 1207–1216. 10.1002/ceat.200301836

[B49] Nüchter M. Ondruschka B. Bonrath W. Gum A. (2004). Microwave assisted synthesis–a critical technology overview. Green Chem. 6, 128–141. 10.1039/b310502d

[B50] Park S.-E. Kim D. S. Chang J.-S. Kim W. Y. (1998). Synthesis of MCM-41 using microwave heating with ethylene glycol. Catal. Today 44, 301–308. 10.1016/S0920-5861(98)00203-X

[B51] Patel S. (2004). Potential of fruit and vegetable wastes as novel biosorbents: Summarizing the recent studies. Rev. Environ. Sci. Biotechnol. 11, 365–380. 10.1007/s11157-012-9297-4

[B52] Pimprom S. Sriboonkham K. Dittanet P. Föttinger K. Rupprechter G. Kongkachuichay P. (2015). Synthesis of copper–nickel/SBA-15 from rice husk ash catalyst for dimethyl carbonate production from methanol and carbon dioxide. J. Ind. Eng. Chem. 31, 156–166. 10.1016/j.jiec.2015.06.019

[B53] Qiao Z.-A. Zhang L. Guo M. Liu Y. Huo Q. (2009). Synthesis of mesoporous silica nanoparticles via controlled hydrolysis and condensation of silicon alkoxide. Chem. Mater. 21, 3823–3829. 10.1021/cm901335k

[B54] Ravandi R. Khoshbin R. Karimzadeh R. (2018). Synthesis of free template ZSM-5 catalyst from rice husk ash and co-modified with lanthanum and phosphorous for catalytic cracking of naphtha. J. Porous Mater. 25, 451–461. 10.1007/s10934-017-0457-3

[B55] Renuka N. K. Praveen A. K. Anas K. (2013). Influence of CTAB molar ratio in tuning the texture of rice husk silica into MCM 41 and SBA-16. Mater. Lett. 109, 70–73. 10.1016/j.matlet.2013.07.074

[B56] Sandoval-Díaz L.-E. Aragon-Quiroz J.-A. Ruíz-Cardona Y.-S. Domínguez-Monterroza A.-R. Trujillo C.-A. (2017). Fractal analysis at mesopore scale of modified USY zeolites by nitrogen adsorption: A classical thermodynamic approach. Microporous Mesoporous Mater. 237, 260–267. 10.1016/j.micromeso.2016.08.030

[B57] Schneider D. Wassersleben S. Weiß M. Denecke R. Stark A. Enke D. (2018). A generalized procedure for the production of high-grade, porous biogenic silica. Waste Biomass Valori. 1–15. 10.1007/s12649-018-0415-6

[B58] Slangen P. M. Jansen J. C. van Bekkum H. (1997). The effect of ageing on the microwave synthesis of zeolite NaA. Microporous Mater. 9, 259–265. 10.1016/S0927-6513(96)00119-8

[B59] Steel A. Carr W. S. Anderson M. W. (1994). ^14^N NMR study of surfactant mesophases in the synthesis of mesoporous silicates. J. Chemisher. Soc. Chem. Commun. 1571–1572. 10.1039/C39940001571

[B60] Thommes M. Kaneko K. Neimark A. V. Olivier J. P. Rodriguez-Reinoso F. Rouquerol J. (2015). Physisorption of gases, with special reference to the evaluation of surface area and pore size distribution (IUPAC Technical Report). Pure Appl. Chem. 87, 1051–1069. 10.1515/pac-2014-1117

[B61] Tsyntsarski B. Stoycheva I. Tsoncheva T. Genova I. Dimitrov M. Petrova B. (2015). Activated carbons from waste biomass and low rank coals as catalyst supports for hydrogen production by methanol decomposition. Fuel Proces. Technol. 137, 139–147. 10.1016/j.fuproc.2015.04.016

[B62] Uhlig H. Gimpel M.-L. Inayat A. Gläser R. Schwieger W. Einicke W.-D. (2013). Transformation of porous glasses into MCM-41 containing geometric bodies. Micropor. Mesopor. Mat. 182, 136–146. 10.1016/j.micromeso.2013.08.035

[B63] Uhlig H. Münster T. Kloess G. Ebbinghaus S. G. Einicke W.-D. Gläser R. (2018). Synthesis of MCM-48 granules with bimodal pore systems via pseudomorphic transformation of porous glass. Micropor. Mesopor. Mat. 257, 185–192. 10.1016/j.micromeso.2017.08.033

[B64] Umeda J. Kondoh K. (2010). High-purification of amorphous silica originated from rice husks by combination of polysaccharide hydrolysis and metallic impurities removal. Ind. Crop. Prod. 32, 539–544. 10.1016/j.indcrop.2010.07.002

[B65] Vandenberghe L. P. S. Soccol C. R. Pandey A. Lebeault J.-M. (2000). Solid-state fermentation for the synthesis of citric acid by Aspergillus niger. Biores. Technol. 74, 175–178. 10.1016/S0960-8524(99)00107-8

[B66] Vempati R. K. Borade R. Hegde R. S. Komarneni S. (2006). Template free ZSM-5 from siliceous rice hull ash with varying C contents. Micropor. Mesopor. Mat. 93, 134–140. 10.1016/j.micromeso.2006.02.008

[B67] Venezia A. M. La Parola V. Longo A. Martorana A. (2001). Effect of alkali ions on the amorphous to crystalline phase transition of silica. J. Solid State Chem. 161, 373–378. 10.1006/jssc.2001.9345

[B68] Voegtlin A. C. Matijasic A. Patarin J. Sauerland C. Grillet Y. Huve L. (1997). Room-temperature synthesis of silicate mesoporous MCM-41-typematerials: Influence of the synthesis pH on the porosity of the materials obtained. Micropor. Mater. 10, 137–147. 10.1016/S0927-6513(97)00003-5

[B69] Wu C.-G. Bein T. (1996). Microwave synthesis of molecular sieve MCM-41. Chem. Commun. 925–926. 10.1039/cc9960000925

[B70] Yahya M. A. Al-Qodah Z. Ngah C. Z. (2015). Agricultural bio-waste materials as potential sustainable precursors used for activated carbon production: A review. Renew. Sustainable Energy Rev. 46, 218–235. 10.1016/j.rser.2015.02.051

[B71] Yan F. Jiang J. Tian S. Liu Z. Shi J. Li K. (2016). A green and facile synthesis of ordered mesoporous nanosilica using coal fly ash. ACS Sustainable Chem. Eng. 4, 4654–4661. 10.1021/acssuschemeng.6b00793

[B72] Yeletsky P. M. Yakovlev V. A. Mel'gunov M. S. Parmon V. N. (2009). Synthesis of mesoporous carbons by leaching out natural silica templates of rice husk. Micropor. Mesopor. Mat. 121, 34–40. 10.1016/j.micromeso.2008.12.025

[B73] Zhang C. Li S. Bao S. (2018). Sustainable synthesis of ZSM-5 zeolite from rice husk ash without addition of solvents. Waste Biomass Valor. 1–11. 10.1007/s12649-018-0356-0

