# Supplementary material for: Low-Cost Microwave-Assisted Partial Pseudomorphic Transformation of Biogenic Silica
Source: Front Chem. 2019 Aug 13;7:575. doi: 10.3389/fchem.2019.00575 (PMC6705224; doi:10.3389/fchem.2019.00575)
Supplement: Supplementary file 1 [file Data_Sheet_1.docx]

Supplementary Material

Low-cost Microwave-assisted Partial Pseudomorphic Transformation of Biogenic Silica

Denise Schneider^1, *^, Ralf Kircheis^1^, Susan Wassersleben^1^, Wolf-Dietrich Einicke^1^, Roger Gläser^1^, Dirk Enke^1^

^1^Universität Leipzig, Institute of Chemical Technology, Linnéstr. 3, 04103 Leipzig, Germany

*** Correspondence:**Denise Schneider
denise.schneider@uni-leipzig.de, ORCID 0000-0002-0450-9176


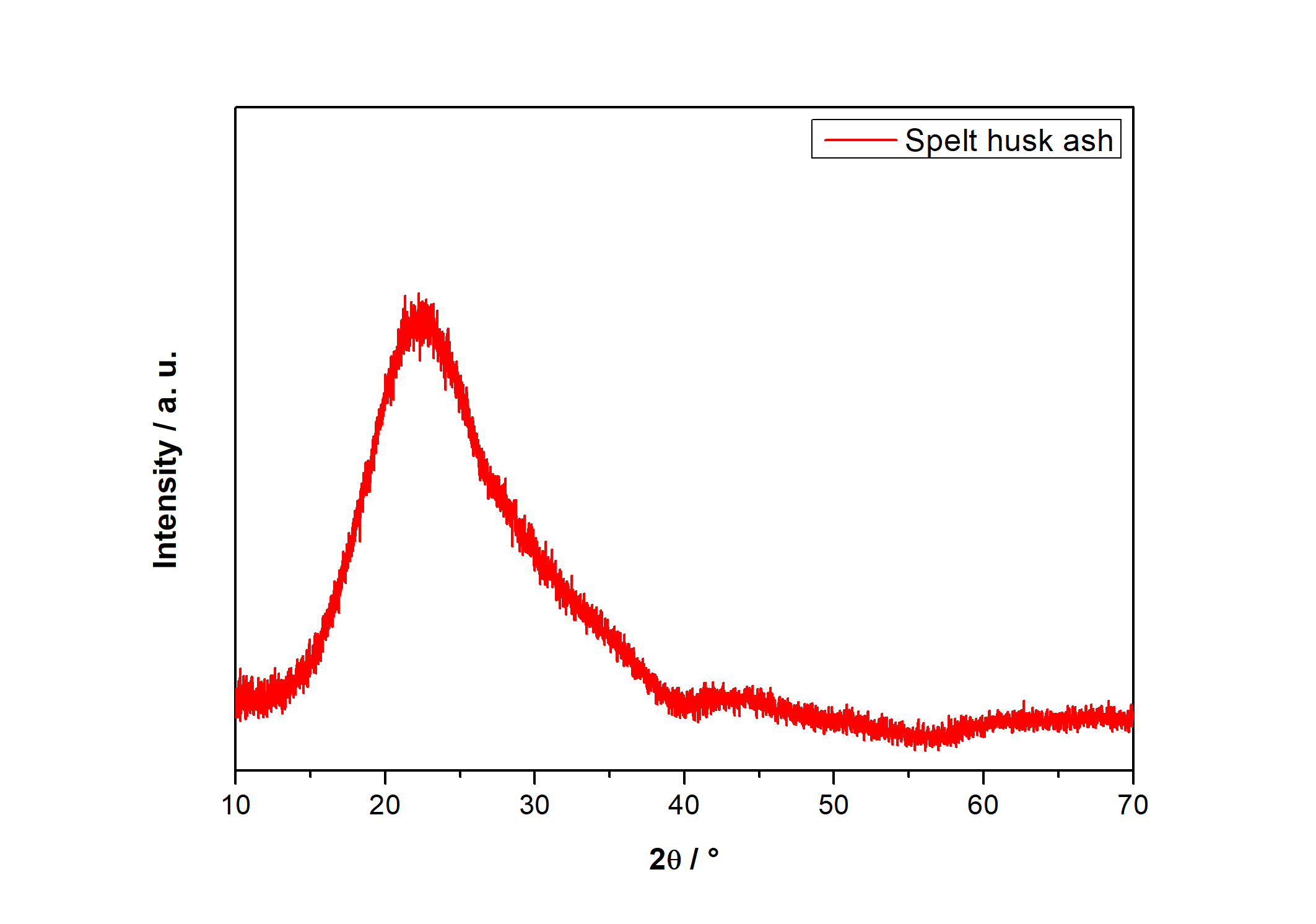


**Supplementary Figure 1.** XRD pattern of the starting material spelt husk ash obtained after leaching and burning.

**Supplementary Table 1.** Textural properties (determined by nitrogen sorption) of HPLC silica and Aerosil^®^ 90 before and after partial transformation in the drying oven for 24 h and in the microwave for 10 min at 393 K with 0.09 M CTACl and 0.09 M NaOH (CTACl + NaOH)

| Sample | Conditions | *S_BET_* / m^2^ g^-1^ | | *V_P_* / cm³ g^-1^ | *d_P_* / nm |
| --- | --- | --- | --- | --- | --- |
| HPLC SiO_2_ | untreated | | 291 | 1.08 | 15.0 |
| HPLC SiO_2_ | CTACl + NaOH: Microwave, 10 min | | 544 | 0.98 | 5.4 |
| HPLC SiO_2_ | CTACl + NaOH: Oven, 24 h | | 763 | 1.00 | 7.2 |
| Aerosil^®^ 90 | untreated | | 90 | - | - |
| Aerosil^®^ 90 | CTACl + NaOH: Microwave, 10 min | | 396 | 0.51 | 5.2 |
| Aerosil^®^ 90 | CTACl + NaOH: Oven, 24 h | | 675 | 0.65 | 3.8 |


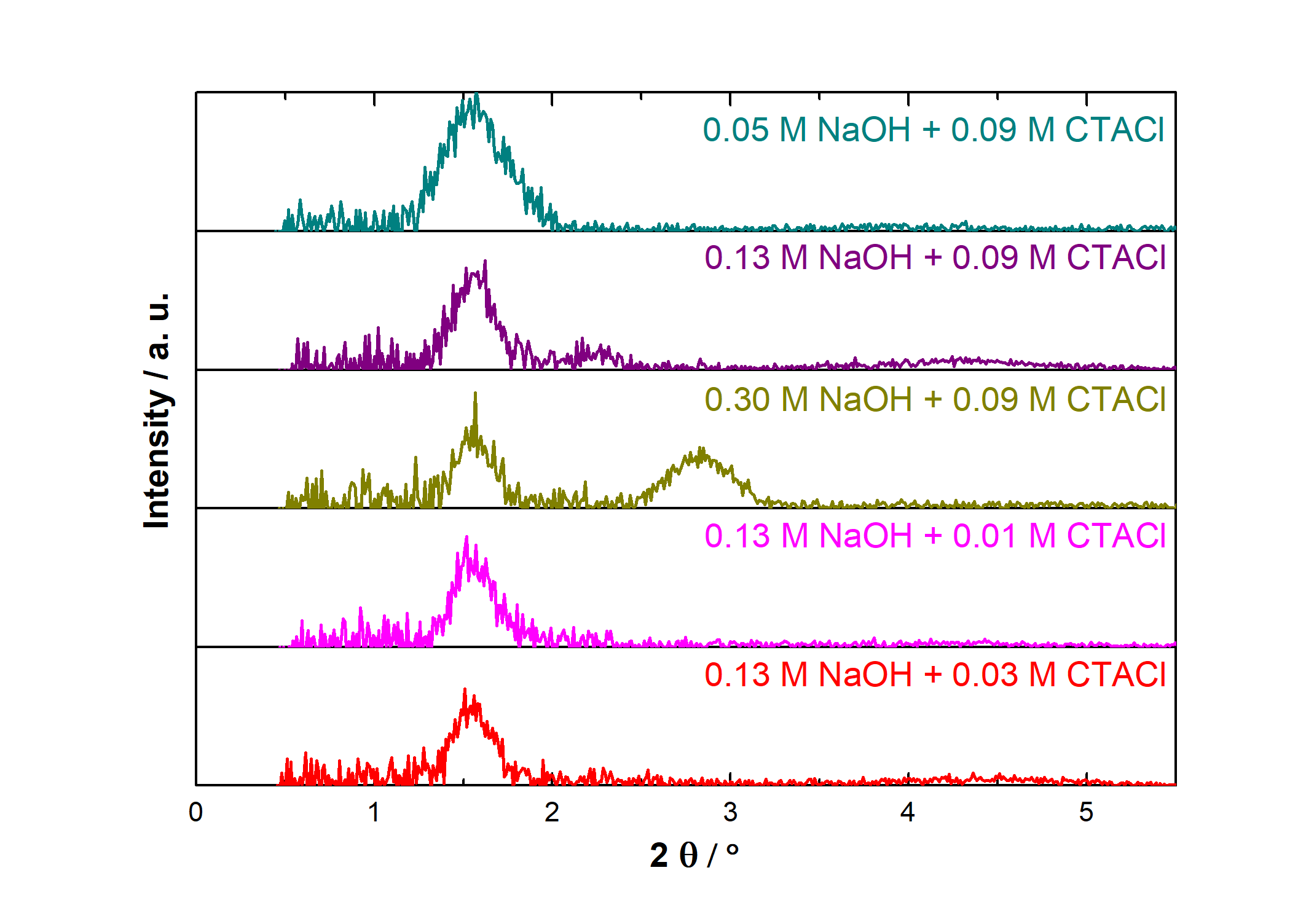


**Supplementary Figure 2.** SAXS patterns of the selected materials after partial transformation in the microwave for 10 min at 393 K with different concentrations of NaOH and CTACl.
